# Mechanism of Neural Regeneration Induced by Natural Product LY01 in the 5×FAD Mouse Model of Alzheimer’s Disease

**DOI:** 10.3389/fphar.2022.926123

**Published:** 2022-06-22

**Authors:** Xiao-Wan Li, Yang-Yang Lu, Shu-Yao Zhang, Ning-Ning Sai, Yu-Yan Fan, Yong Cheng, Qing-Shan Liu

**Affiliations:** ^1^ Key Laboratory of Ethnomedicine for Ministry of Education, Center on Translational Neuroscience, Minzu University of China, Beijing, China; ^2^ Center for Life Sciences, School of Life Science and Technology, Harbin Institute of Technology, Harbin, China; ^3^ University Hospital, Tianjin Normal University, Tianjin, China; ^4^ Traditional Chinese Medicine Department, Beijing Tiantan Hospital, Capital Medical University, Beijing, China; ^5^ Institute of National Security, Minzu University of China, Beijing, China

**Keywords:** isoflavone-cytisine, Alzheimer’s disease, 5×FAD mice, neural stem cells, neural regeneration

## Abstract

**Background:** A sharp decline in neural regeneration in patients with Alzheimer’s disease (AD) exacerbates the decline of cognition and memory. It is of great significance to screen for innovative drugs that promote endogenous neural regeneration. Cytisine N-methylene-(5,7,4′-trihydroxy)-isoflavone (LY01) is a new compound isolated from the Chinese herbal medicine *Sophora alopecuroides* with both isoflavone and alkaloid characteristic structures. Its pharmacological effects are worth studying.

**Objective:** This study was designed to determine whether LY01 delays the cognitive and memory decline in the early stage of AD and whether this effect of LY01 is related to promoting neural regeneration.

**Methods:** Eight-week-old 5×Familial Alzheimer’s Disease (5×FAD) mice were used as disease models of early AD. Three doses of LY01 administered in two courses (2 and 5 weeks) of treatment were tested. Cognition, memory, and anxiety-like behaviors in mice were evaluated by the Morris water maze, fear conditioning, and open field experiments. Regeneration of neurons in the mouse hippocampus was observed using immunofluorescence staining. The effect of LY01 on cell regeneration was also demonstrated using a series of tests on primary cultured neurons, astrocytes, and neural stem cells (NSCs). In addition, flow cytometry and transcriptome sequencing were carried out to preliminarily explored the mechanisms.

**Results:** We found that LY01 reduced the decline of cognition and memory in the early stage of 5×FAD mice. This effect was related to the proliferation of astrocytes, the proliferation and migration of NSCs, and increases in the number of new cells and neural precursor cells in the dentate gyrus area of 5×FAD mice. This phenomenon could be observed both in 2-week-old female and 5-week-old male LY01-treated 5×FAD mice. The neuronal regeneration induced by LY01 was related to the regulation of the extracellular matrix and associated receptors, and effects on the S phase of the cell cycle.

**Conclusion:** LY01 increases the proliferation of NSCs and astrocytes and the number of neural precursor cells in the hippocampus, resulting in neural regeneration in 5×FAD mice by acting on the extracellular matrix and associated receptors and regulating the S phase of the cell cycle. This provides a new idea for the early intervention and treatment of AD.

## Introduction

Alzheimer’s disease (AD) is a progressive neurodegenerative disease (Berchtold and Cotman, 1998). According to the data published by the Alzheimer’s Disease International association in 2015, there are more than 54.4 million patients with dementia worldwide, with an average increase of one case every 3 s (Dos Santos Picanco et al., 2018), and most of them are related to AD (Bradley et al., 2015; Livingston et al., 2017; Alzheimer’s Association, 2020). The most recent data indicate that, by 2050, the prevalence of dementia will triple worldwide (Scheltens et al., 2021), and that estimate is three times higher when based on a biological (rather than clinical) definition of AD. The main pathological features (Zilka and Novak, 2006; Cipriani et al., 2011) of AD are extracellular amyloid plaques, intracellular neurofibrillary tangles, and loss of neurons (Blennow et al., 2006).

Neural regeneration, including the proliferation and functional differentiation of NSCs, is a process in which neural networks are established and maintained to function well through continual plastic changes and establishing synaptic connections with other neurons. Adult NSCs have the ability to self-renew and differentiate into neurons and glial cells. In the developed nervous system, NSCs derived from embryonic cells carried on the neural plate can exist in the adult hippocampus and lateral ventricle for a long time. The identity of NSCs between pluripotent state and post differentiation state is regulated by the response of the NSCs transcription program under the influence of external and internal factors. External factors and internal factors act at the same time, endowing the central nervous system (CNS) with different levels of plasticity and necessary complexity (Massirer et al., 2011). Studies using human brain samples taken under strict conditions and advanced tissue processing technology revealed that adult hippocampal nerve regeneration is very rich in healthy subjects, but decreases sharply in patients with AD (Moreno-Jimenez et al., 2019). Other studies have pointed out that the interactions between sleep disorders and inhibition of nerve regeneration exacerbate the cognitive decline associated with AD (Kent and Mistlberger, 2017). Recovery of the number of new stem cells in the brain of AD patients is of great value for the improvement of the disease condition (Wareham et al., 2022). Therefore, the research on NSC therapy for AD has persisted (Hayashi et al., 2020; Aboul-Soud et al., 2021). However, the costs of the operation are high and the compliance of patients with targeted transplantation is poor. Therefore, alternatives, such as active compounds that can promote the proliferation and migration of endogenous NSCs in the CNS, are needed.

**FIGURE1:**
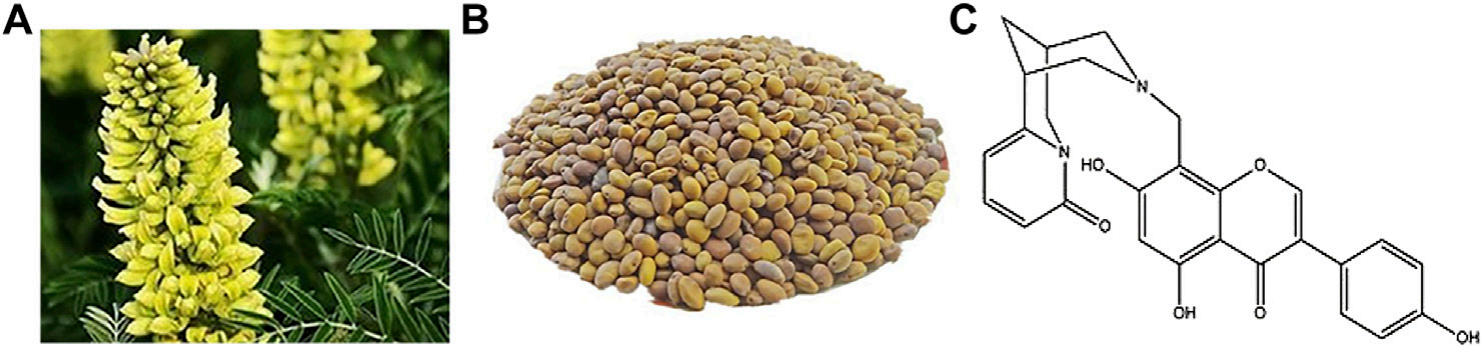
Molecular structure of LY01.

Cytisine N-methylene-(5,7,4′-trihydroxy)-isoflavone is a new compound isolated from the Chinese herbal medicine *Sophora alopecuroides*. It is a new type of cytisine with an alkaloid and isoflavone structure (Figure 1) (Yin and Liu, 2016). Alkaloids are an important type of secondary metabolites in plants. They usually have significant pharmacological activities and biological functions and have rich chemical structures in nature. Known studies have found that the activities of alkaloids are diverse, including anti-tumor (Manayi et al., 2018), antibacterial (de Oliveira et al., 2019), blood glucose-regulating (Li et al., 2017), and antiviral (Varghese et al., 2016) activities. Other studies have shown that alkaloids can selectively bind to and serve as an agonist of the α4β2 nicotinic acetylcholine receptor, thus inhibiting addiction. It has been used in the study of smoking cessation (Rigotti, 2014), which indicates the potential value of alkaloids in the treatment of nervous system diseases. Flavones have many functions. They are a strong antioxidants whose capacities are more than 10 times that of vitamin E. They effectively remove oxygen free radicals in the body (Popovic et al., 2019), prevent cell degeneration and aging, and prevent cancer (Mazewski et al., 2018; Chen et al., 2020); they also improve blood circulation and lower cholesterol (Singh et al., 2018), inhibit the exudation of inflammatory enzymes, promote wound healing, and relieve pain (Shoaib et al., 2019). Isoflavone is a flavonoid that mainly exists in leguminous plants. It is called phytoestrogen because of its structural similarity to estrogen, which can be used for the prevention and even treatment of Alzheimer’s disease (Duan et al., 2021). Phytoestrogens are not pharmaceutical estrogens and have few side effects (Escande et al., 2006)**,** and have a two-way regulation function. When the estrogen in the body is high, they show anti-estrogen activity and reduce the risk of related cancers. As a new compound with both alkaloid and isoflavone active structures, there has been no report on the biological activity of LY01 before. This research on the treatment of AD with the bioactive substance LY01 will provide a new choice for the prevention and treatment of the disease. Likewise, the research on the biological activities of LY01 related to neurons, astrocytes, and NSCs will further enrich the understanding of the mechanism of action of isoflavones.

## Materials and Methods

### Primary Cell Culture and Treatment

Primary astrocytes (Chen et al., 2019), neurons (Pan et al., 2017)**,** and NSCs (Wei et al., 2020) were cultured according to the previous protocols. Primary hippocampal neurons were evenly spread into 96-well plates at the concentration of 5×10^5^ cells/mL. On the third to fifth day of culture *in vitro*, LY01 (3, 6, 12, 24, 48 μm) treatment was performed when the morphology and structure of neurons were relatively mature. Cell viability was measured at 48 h after the cells were incubated with LY01.

Purified astrocytes were evenly spread into 96-well plates at the concentration of 1×10^5^ cells/mL. The media was changed after 12 h of culturing and treated with LY01 (1.5, 3, 6 μm). After 48 h, cell viability was measured. Three replicates were set up and the experiment was repeated three times independently.

The primary NSCs with good proliferation and property were evenly spread into 96 well plate and 6-well plate at the concentration of 1×10^5^ cells/mL. LY01 treatment (0.75, 1.5, 3, 6 μm) was carried out 24 h after plating cells. The cell viability, neurospheres number and gene expression level of laminin subunit gamma 2 (Lamc2) were measured 24 h after the treatment. The cells in the 6-well plate were collected for cell cycle detection. In addition, the primary NSCs digested into single cells were evenly plated on coverslips coated with poly-l-lysine at the concentration of 2×10^4^ cells/mL. After 12 h, NSC proliferation medium was changed into NSC differentiation medium (Dulbecco’s modified Eagle’s medium (DMEM)/F12 medium containing 10% fetal bovine serum (FBS)) and cultured for 7 days. LY01 was added to the differentiation medium at the concentration of 1.5 μm and the medium was changed every other day. The differentiated cells was detected by immunofluorescence. Images were randomly captured for quantification.

The experiments were repeated three times independently. LY01 was dissolved in DMSO and basic medium, and the final concentration of DMSO was less than 1‰. The control group was treated with the same amount of vehicle as the experimental group.

### Cell Viability Test

Cell viability was detected by water-soluble tetrazolium salt 1 (WST1) kit (Beyotime Biotechnology, C0036) according to the previous protocols (Wei et al., 2020).

### Neurospheres Counting

After treatment, NSC neurospheres were counted in the bright field of fluorescence microscope. Three replications were set for each independent experiment, and four images were randomly captured in each replication for neurospheres counting. The image Pro software was used for statistical analysis. The experiment was repeated three times independently.

### Transwell Migration Assay

The primary NSCs with good properties were prepared into a single cell suspension of 1×10^6^ cells/mL in serum-free DMEM/F12 medium. The volume of cell suspension in the upper chamber of transwell was 100 μL. 600 μL DMEM/F12 medium containing 10% FBS supplemented with or without 1.5 μΜ LY01 was added into the bottom wells, followed by incubation for 12 h. The medium in the upper chamber was discarded, and 800 μL phosphate buffer (PBS) was used to gently clean the upper membrane twice. After that, 800 μL methanol was uesd to fix the cells on the membrane for 30 min at room temperature. After staining with 0.1% crystal violet solution for 20 min at room temperature, the nonmigrating cells on the upper membrane were gently removed with a cotton swab. Images were randomly captured, and the number of migrating cells were manually quantified. The experiment was repeated three times independently.

### Morphological Analysis of Primary NSC Migration

On the fourth day of primary NSC passage, the neurospheres were collected and plated to 24 well plate coated with poly-l-lysine. After 4 h, 1.5 μm LY01 was administered, and then cultured for 19–24 h 10 neurospheres were randomly captured from each group, and The ratio of the diameter of the radiation circle after migration to the diameter of the protoneurospheres was used to reflect the effect of LY01 on cell migration. The experiment was repeated three times.

### Cell Cycle Detection

After centrifugation at 800 g for 5 min, neurospheres were collected and digested, and then the cells were washed twice with precooled PBS. Each sample was added with 500–1000 μL precooled 70% ethanol, and the cell suspension concentration was 0.5–2×10^6^ cells/mL, followed by being fixed overnight at 4°C or long-term storage at −20°C. The cells were collected by centrifugation, washed with 1 ml of precooled PBS once, and then resuspended with 500 μL PBS containing 50 μg/ml propidium iodide (Solarbio, P8080), 100 μg/ml RNase A, 0.2% Triton X-100, and incubated in dark at 4°C for 30 min. The samples were detected by FlowSight multi-dimensional panoramic flow cytometry, and cell cycle was analyzed by IDEAS^®^. Three replications were set in each group.

### RNA Sequencing

NSCs were plated into a 12 well plate at the concentration of 1×10^5^ cells/mL. After 24 h, the cells were divided into two groups: control group treated with vehicle and experimental group treated with 1.5 μm LY01 for 6 h. The cells were resuspended with 1 ml Trizol and then frozen at −80°C for testing. Samples prepared according to the above steps were sent to the Shanghai Majorbio Bio-pharm Technology Co., Ltd. for RNA extraction, illumina transcriptome sequencing and bioinformatics analysis (Ren et al., 2022).

### RNA Extraction and Quantitative RT-PCR

RNA was extracted from primary NSCs using Trizol reagent (ThermoFisher, Waltham, MA, United States) and converted to cDNA using TaqManTM MicroRNA Reverse Transcription kit (ThermoFisher) following the manufacturer’s protocol. The quantitative reverse transcriptase-polymerase chain reaction (RT-PCR) were performed2×RealStar Fast SYBR qPCR Mix (Genestar, A301-10). The cycling c-onditions were: 2 min denaturation at 95°C and 45 cycles of DNA synthe-sis at 95°C for 15 s and 60°C for 30 s. Primer sequences for Lamc2 forward: 5′-GCA​TCT​ACA​ACA​CAG​CGG​GAA-3′, reverse: 5′-ACA​GCT​GCC​ATC​ACT​TCG​AC-3’; for GADPH forward: 5′-ATC​AAC​GGG​AAA​CCC​ATC​ACC-3′, reverse: 5′-AAG​ACG​CCA​GTA​GAC​TCC​AC-3’. All quantitative RT-PCR reactions were performed using a LightCycler^®^ 96 Real-Time PCR Detection System (Roche, Basel, Switzerland).

### Animals

The 5×FAD mice overexpressing the K670 N/M671 L (Swedish), I716V (Florida), and V717I (London) mutations in human APP, as well as M146 L and L286 V mutations in human PS1, were provided by Z. Q Zhang (Beijing Institute of Basic Medical Sciences, Beijing, China). Genotypes were confirmed by PCR analysis of tail biopsy specimens. Mice were housed four to five per cage with a 12-h light/12-h dark cycle and food and water adlibitum. All experimental animal procedures were approved by the Institutional Animal Care and Use Committees of the Minzu University of China.

### Drug Treatment

**FIGURE 2:**
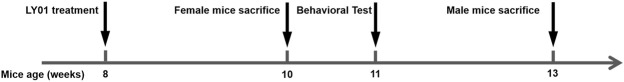
Schedule for drug administration and behavioral testing.

The experiment was divided into six groups, wild type (WT) group, 5×FAD group, Rg1 group, and LY01-high, middle, low group. Except WT group, other groups were 5×FAD mice. This experiment mainly focused on the effect of LY01 in the early stage of AD, so the drug was administered at the age of 8 weeks when 5×FAD mice exhibit amyloid deposition (Eimer and Vassar, 2013; Gu., 2015) and the rate of hippocampal neural regeneration changed relatively little with age (Harris et al., 2021). The age difference was within 5 days for female mice, and 7 days for male mice. The dosage of Rg1 group was 20 mg/kg/day, while that of LY01 was 0.025, 0.1 and 0.4 mg/kg/day, respectively. The other two groups were intraperitoneally injected with the same amount of normal saline every day. WT mice used in the experiment were siblings of 5×FAD mice. All the mice weighed in the range of 20 ± 2 g (g), and were randomly divided into each group, and there was no significant difference in body weight between groups. Under the advocacy of the 3R (Reduction, RepIacement, Refinement) principle of animal experiment, two different courses of treatment were used for female and male mice to get more information about the efficacy of the new drugs in the course of treatment and gender, rather than their differences, with as few animals as possible. As pathological characteristics of 5×FAD mice were more obvious at the age of 3 months (Jawhar et al., 2012), the AD-like behavioral phenotype should be more significant at this age. In addition, compared with female mice, male mice are less disturbed by the fluctuation of hormone level, and have better physical strength for water maze experiment (Pan et al., 2017). Therefore, a 5-weeks treatment course was set for male mice, the sample size of each group was no less than eight mice, and for female mice, a 2-weeks course (Liu et al., 2021) of treatment was used, the sample size of each group was no less than three mice. The mice were intraperitoneally injected with BrdU solution at a dose of 50 mg/kg/day for three consecutive days starting from the fourth days before sampling (Figure 2).

### Morris Water Maze

Water maze is a classical experimental method to test the learning and memory ability of experimental animals. The experimental site is divided into four quadrants. In one quadrant, a transparent platform can be set for mice to stand. Once the platform position is set, it cannot be changed during the experiment. At the same time, a graphic mark can be made around the experimental pool for reference. Before the experiment, diluted milk should be added to the pool to make the water turbid. The temperature of water requires constant temperature during the experiment, which is generally 18–22°C. The experiment is divided into two stages, positioning navigation and space exploration. The training days of the positioning navigation experiment were 6 days, and the single training time was 60 s each mouse was put into the water from four quadrants to find the platform in the water. The time used in this process is called the escape latency. If the mice cannot find the platform within the specified time, they will stay for 10 s after arriving at the platform to deepen learning. On the seventh day, the platform was removed, and the movement of mice within 60 s was recorded.

### Fear Conditioning

Fear conditioning is a common experimental method to test the conditioned memory ability of animals. A box is used as the isolation environment for behavioral test. There are 2 W incandescent lamps installed in it, and small exhaust fan is used for ventilation. The bottom of the box is stainless steel bar for foot electrical stimulation, and the top is equipped with loudspeaker for sound stimulation. After the test, the training arena was wiped with alcohol to eliminate the interference of odor on the experimental results. On the first day of the experiment, the mice were put into the box. After 60 s of adaptation, 75 dB white noise stimulation lasting for 30 s was given at 110, 160, 210, 260, 310, 360, 410, 460 s respectively, and foot shock (30 mA) lasting for 2 s was given at the last 2 s of each white noise stimulation. The whole experiment lasted 490 s, and the animal’s freezing time was observed by camera. On the second day of the experiment, the animals were placed in the experimental box for 120 s without any sound or electrical stimulation. The animal’s freezing time was detected through the camera. On the third day of the experiment, the color of the test box was changed with colored paper. After 30 s of adaptation time, white noise (75 dB) stimulation lasting for 30 s was given at 30, 80, 110, 160, 210 s. The whole experiment lasted 150 s, and the animal’s freezing time was recorded.

### Open Field Test

The open field test is used to evaluate the state of autonomic movement, aiming to identify agitation, and pathological behavior. The experimental device consists of an trial chamber, an automatic data acquisition and processing system. The open field lighting is all artificial lighting. The laboratory personnel, computers and other equipment are located in another room to reduce the interference to animals. The background noise of the laboratory is controlled below 65 dB. The mouse trial chamber is 25–30 cm high, the bottom edge is 72 cm long, the inner wall is blackened, and the bottom surface (software) is divided into 64 small squares on average. The rearing times, movement speed, central movement time and other information of mice were recorded within 5 min.

### Immunofluorescence

Specific proteins in cells and tissues were labeled with fluorescence according to the previous experimental protocols (Wei et al., 2020).

For immunofluorescent staining, the cells plated on the coverslips and brain sections were labeled with primary antibody and fluorescent secondary antibodies as follows: GFAP antibody (Millipore, MAB360, 1:1000), MAP2 antibody (CST, 8707S, 1:1000), Ki67 antibody (Invitrogen, 12H15L5, 1:500), DCX antibody (CST, 4604S, 1:2000), BrdU antibody (CST, 5292, 1:800), Donkey anti-Mouse IgG (Invitrogen, A-21202, 1:2000), Donkey anti-Rabbit IgG (Invitrogen, A-21207, 1:2000). For the primary cell experiment, pictures were taken randomly from four areas in each group according to the upper, lower, left and right positions, and take the mean for statistical analysis (Pan et al., 2017; Wei et al., 2020). For animal experiments, continuous slices of the whole hippocampus were made with a thickness of 30 microns. Three slices were selected randomly and evenly, and the positions of brain slices selected in each group were the same. BrdU-positive cells in the dentate gyrus were counted and the mean was taken for statistical analysis (Cheng et al., 2015; Wei et al., 2020).

### Statistical Analysis

All statistical analyses were performed using GraphPad Prism version 6.0 software. Outliers were eliminated by the statistical elution method (values that deviate from the mean ± 2 times the standard deviation are excluded) (Nakagawa and Cuthill, 2007). The significance of differences was assessed by unpaired Student’s t test or one-way ANOVA analysis. The significant threshold was set at *p* < 0.05. The data before analysis of variance were subject to a normality test (D'Agostino-Pearson omnibus test) and variance homogeneity test (Brown-Forsythe test). If not, a nonparametric test was used. For one-way ANOVA analyses, post hoc comparisons were performed using the Bonferroni post hoc tests test. In this paper, the data of *in vitro* experiment were analyzed by *t*-test, and the data of *in vivo* experiment were analyzed by one-way ANOVA.

## Results

### Effect of LY01 on Mouse Body Weight

**FIGURE 3:**
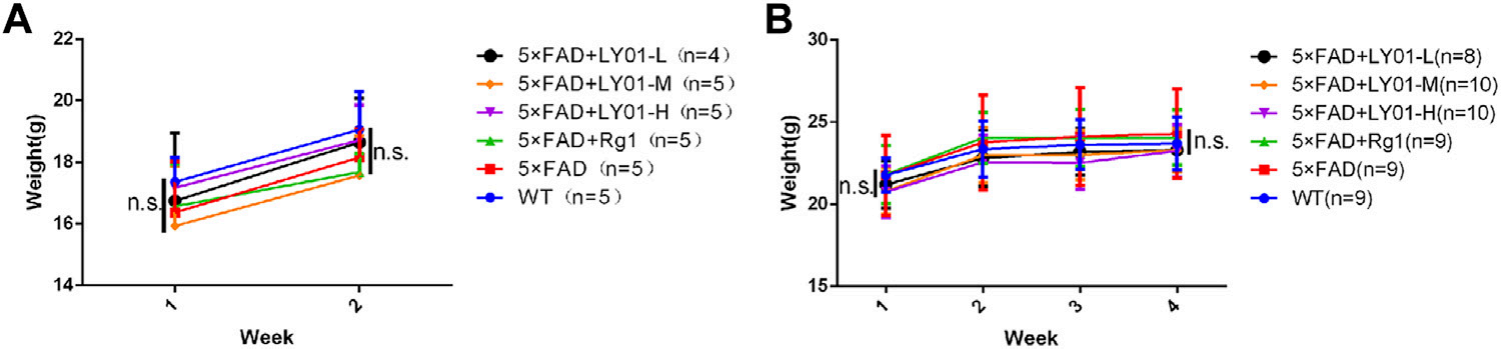
Body weight changes of mice in each group **(A)** Body weight trend of female mice. WT: the control group of wild type mice treated with physiological saline solution for 2 weeks; 5×FAD: the model group of 5×FAD mice treated with physiological saline solution for 2 weeks; 5×FAD + Rg1: the positive control group of 5×FAD mice treated with 20 mg/kg/day ginsenoside Rg1 for 2 weeks; 5×FAD + LY01-H: the experimental group of 5×FAD mice with treated with 0.4 mg/kg/day LY01 for 2 weeks; 5×FAD + LY01-M: the experimental group of 5×FAD mice treated with 0.1 mg/kg/day LY01 for 2 weeks; 5×FAD + LY01-L: the experimental group of 5×FAD mice treated with 0.025 mg/kg/day LY01 for 2 weeks *n* = 4–5 **(B)** Body weight trend of male mice. WT: the control group of wild type mice treated with physiological saline solution for 5 weeks; 5×FAD: the model group of 5×FAD mice treated with physiological saline solution for 5 weeks; 5×FAD + Rg1: the positive control group of 5×FAD mice treated with 20 mg/kg/day ginsenoside Rg1 for 5 weeks; 5×FAD + LY01-H: the experimental group of 5×FAD mice treated with 0.4 mg/kg/day LY01 for 5 weeks; 5×FAD + LY01-M: the experimental group of 5×FAD mice treated with 0.1 mg/kg/day LY01 for 5 weeks; 5×FAD + LY01-L: the experimental group of 5×FAD mice treated with 0.025 mg/kg/day LY01 for 5 weeks *n* = 8–10. n. s, no significance. The results are expressed as means ± SD.

The mice were administered LY01 starting at the age of 8 weeks. During the experiment, body weights of mice was recorded and monitored every week. The results (Figures 3A,B) showed that the body weight of female and male mice increased in the first 2 weeks of the experiment, but there was no significant difference in body weight between the groups. Two weeks after the experiment, the overall increase rate of body weight of male mice slowed down, but there was no significant difference in body weight between the groups. The experimental results show that LY01 did not cause side effects sufficient to affect the weight of mice and can assist in excluding the influence of the health status of mice on the experimental results.

### LY01 Alleviates Cognitive and Memory Decline in AD Mice

**FIGURE 4:**
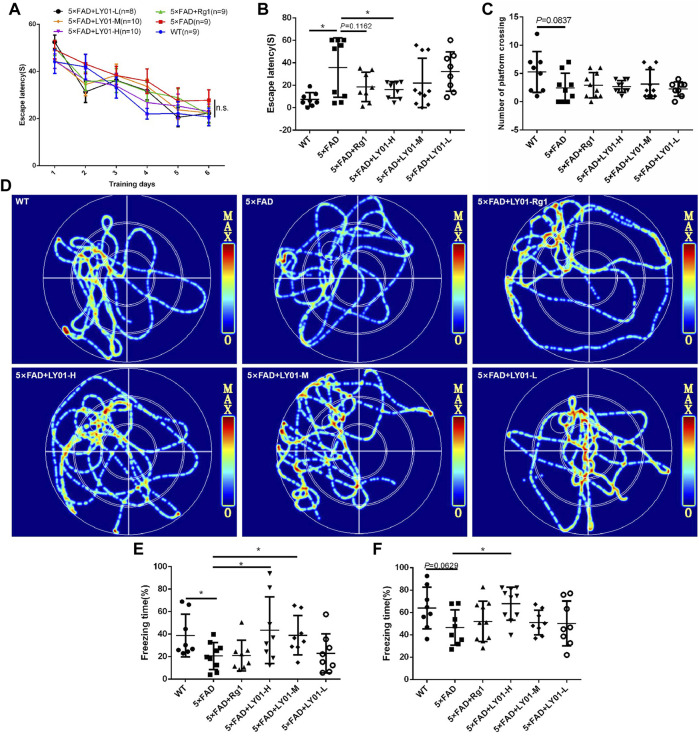
The results of behavioral tests **(A)** Escape latency to the platform during the training trails in the Morris water maze **(B)** Escape latency on the seventh day in the Morris water maze. *p < 0.05 vs. 5×FAD, *n* = 8–10 **(C)** Platform crossings on the seventh day in the Morris water maze. *n* = 8–10 **(D)** Representative track images of mice on the seventh day in the Morris water maze **(E)** Spatial conditioned memory of mice 24 h after training in the fear conditioning test **(F)** conditioned memory ability of mice 48 h after training in the fear conditioning test. WT: the control group of wild type mice treated with physiological saline solution for 5 weeks; 5×FAD: the model group of 5×FAD mice treated with physiological saline solution for 5 weeks; 5×FAD + Rg1: the positive control group of 5×FAD mice treated with 20 mg/kg/day ginsenoside Rg1 for 5 weeks; 5×FAD + LY01-H: the experimental group of 5×FAD mice treated with 0.4 mg/kg/day LY01 for 5 weeks; 5×FAD + LY01-M: the experimental group of 5×FAD mice treated with 0.1 mg/kg/day LY01 for 5 weeks; 5×FAD + LY01-L: the experimental group of 5×FAD mice treated with 0.025 mg/kg/day LY01 for 5 weeks. The results are expressed as means ± SD.

The Morris water maze is a classic behavioral experiment used to test learning and memory in mice. The mice were administrated LY01 intraperitoneally every day starting at the age of 8 weeks and behavior was analyzed after 3 weeks Figure 4A shows the curve of the latency to find the target platform during the training of each group of mice. The results show that the escape latency of mice in each group decreased with the increase in training times, indicating that the training of mice was effective. In addition, except for the model 5×FAD group, the latency of the other groups converged to a minimum value. Although there was no significant difference between the groups, the memories of the three groups and the positive drug Rg1 group tended to appear to be enhanced. According to Figure 4D, the trajectories of mice in the 5×FAD mice group are disordered, and those in the WT group, Rg1 group, and LY01 high-dose administration group are enriched in the target quadrant. As shown in Figure 4B, the space exploration experiment showed that the escape latency of the 5×FAD mice group was much longer than that of the WT group (*p* < 0.05), indicating the success of modeling AD. The escape latency of the LY01 high-dose administration group was significantly lower than that of the 5×FAD mice group (*p* < 0.05). For the Rg1 administration group, there was a downward trend in data distribution compared with the model group (*p* = 0.1162). Overall, these results showed that LY01 delayed the decline of spatial memory in the early stage of 5×FAD mice. Figure 4C shows the number of mice in each group crossing the platform in the space exploration experiment. There is no significant difference between the WT group and the 5×FAD group, but a trend of difference (*p* = 0.0837), and there is no significant difference in other groups compared with the model group. The evaluation index (the number of platform crossings) does not correspond well to the memory decline at the early stage of 5×FAD mice.

In the fear conditioning experiment, environmental and sound stimulation were used to establish a connection with electric shock. Spatial conditioned memory and sound conditioned memory were evaluated using whole-body freezing caused by fear of electric shock. Figure 4E shows the short-term conditioned memory of mice after learning for 24 h, in which there is a significant downward trend in freezing time in 5×FAD groups compared with the WT group (*p* < 0.05), indicating the success of the model. In addition, compared with the model group, the conditioned memory of the high-dose and medium-dose LY01 group increased (*p* < 0.05), indicating that LY01 is beneficial for the improvement of short-term conditioned memory ability in 5×FAD mice. Figure 4F shows the conditioned memory ability of mice 48 h after learning. For this test, the difference between the model group and high-dose LY01 group was significant (*p* < 0.05), indicating that a high dose of LY01 can improve the conditioned memory ability of 5×FAD mice. However, the conditioned memory ability 48 h after training does not reflect the early memory decline of 5×FAD mice at the early stage (*p* = 0.0629).

In the open field test, the mice successively entered the experimental field independently. The mice in each group mainly focused their activities in the surrounding area, with many standing times and a little activity time in the central area (Supplementary Figure S1A), showing obvious exploratory behavior and excitement. There was no significant difference in movement speed and rearing time between the model group and the LY01-administered group (Supplementary Figures S1B,C). The analysis of the open field test results shows that there were no significant differences in exercise ability and anxiety-like behavior at the early stage of 5×FAD mice compared with WT mice, and LY01 did not affect exercise ability and anxiety-like behavior in AD model mice.

### LY01 Could Increase the Number of New Cells in Dentate Gyrus Area of 5×FAD Mice

**FIGURE 5:**
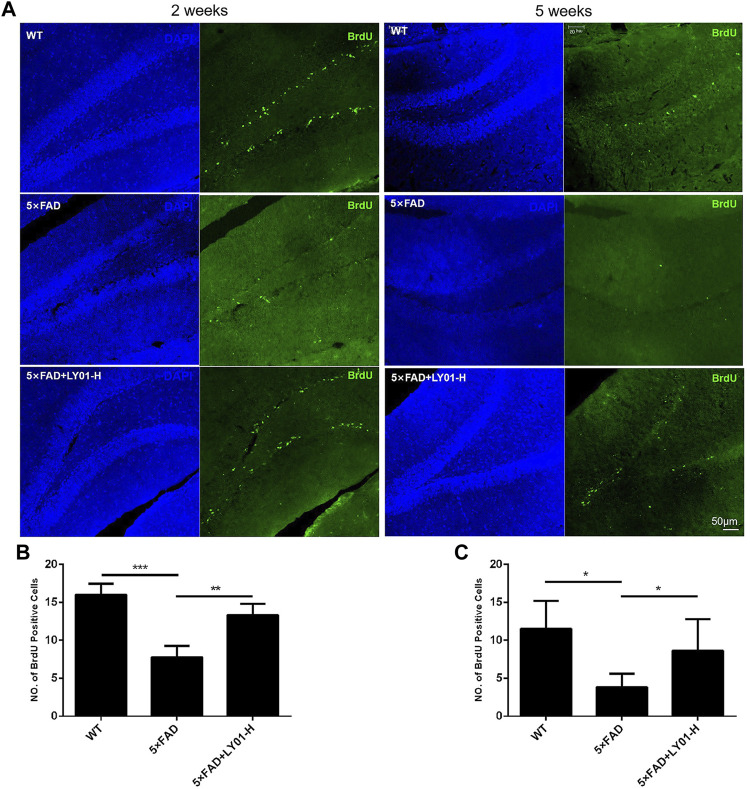
The effect of LY01 on the number of new cells in the dentate gyrus area of 5×FAD mice **(A)** Representative images of new cells in the dentate gyrus of the hippocampus. The images show the result of female mice treated for 2 weeks and male mice treated for 5 weeks. The nuclei are labeled with 4′,6-diamidino-2-phenylindole (DAPI) (blue) and new cells are labeled with BrdU antibody (green) **(B)** The number of new cells in the dentate gyrus of female mice treated for 2 weeks **p < 0.01, ***p < 0.001 vs. 5×FAD; *n* = 3 **(C)** The number of new cells in the dentate gyrus of male mice treated for 5 weeks *p < 0.05 vs. 5×FAD; *n* = 4. WT: the control group of wild type mice treated with physiological saline solution; 5×FAD: the model group of 5×FAD mice treated with physiological saline solution; 5×FAD + LY01-H: the experimental group of 5×FAD mice treated with 0.4 mg/kg/day LY01. The results are expressed as means ± SD.

Bromodeoxyuridine (BrdU) is a thymine nucleoside analogue that can replace thymine to infiltrate DNA molecules in the process of cell proliferation, thus marking new cells. According to the results shown in Figures 5A,B, the number of new cells in the dentate gyrus area of female 5×FAD mice treated for 2 weeks in the group treated with the high concentration of LY01 increased significantly compared with that in the model group (*p* < 0.01). In male 5×FAD mice treated for 5 weeks, the same phenomenon was observed in the high-dose group (Figure 5C, *p* < 0.05). In addition, the number of new cells in 10-week-old female and 13-week-old male 5×FAD mice was significantly lower than that in the WT group (*p* < 0.05).

**FIGURE 6:**
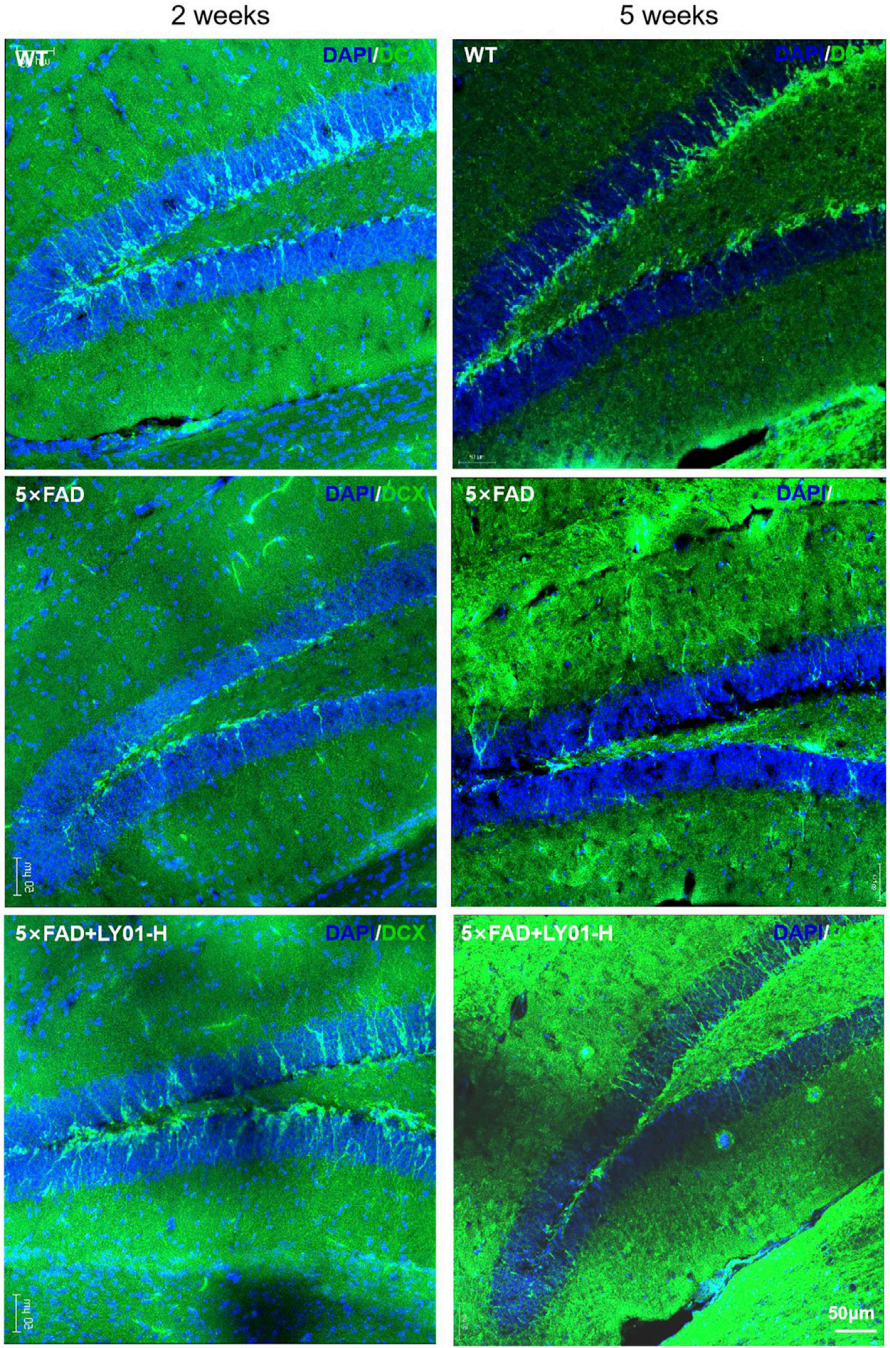
Representative images of neural precursor cells in the dentate gyrus area of 5×FAD mice. The images show the result of female mice treated for 2 weeks and male mice treated for 5 weeks. The nuclei are labeled with DAPI (blue) and neural precursor cells are labeled with doublecortin (DCX) antibody (green).

Doublecortin (DCX) is a marker of neuronal precursor cells. Immunofluorescence staining results of both 10-week-old female and 13-week-old male mice showed that the number of neuronal precursor cells in 5×FAD mice was small and the cell processes were short. Mice administered high-dose LY01 had a higher number of neuronal precursor cells and longer cell neurites than those not treated with LY01 (Figure 6).

### LY01 Promotes the Proliferation of Primary Astrocytes

**FIGURE 7:**
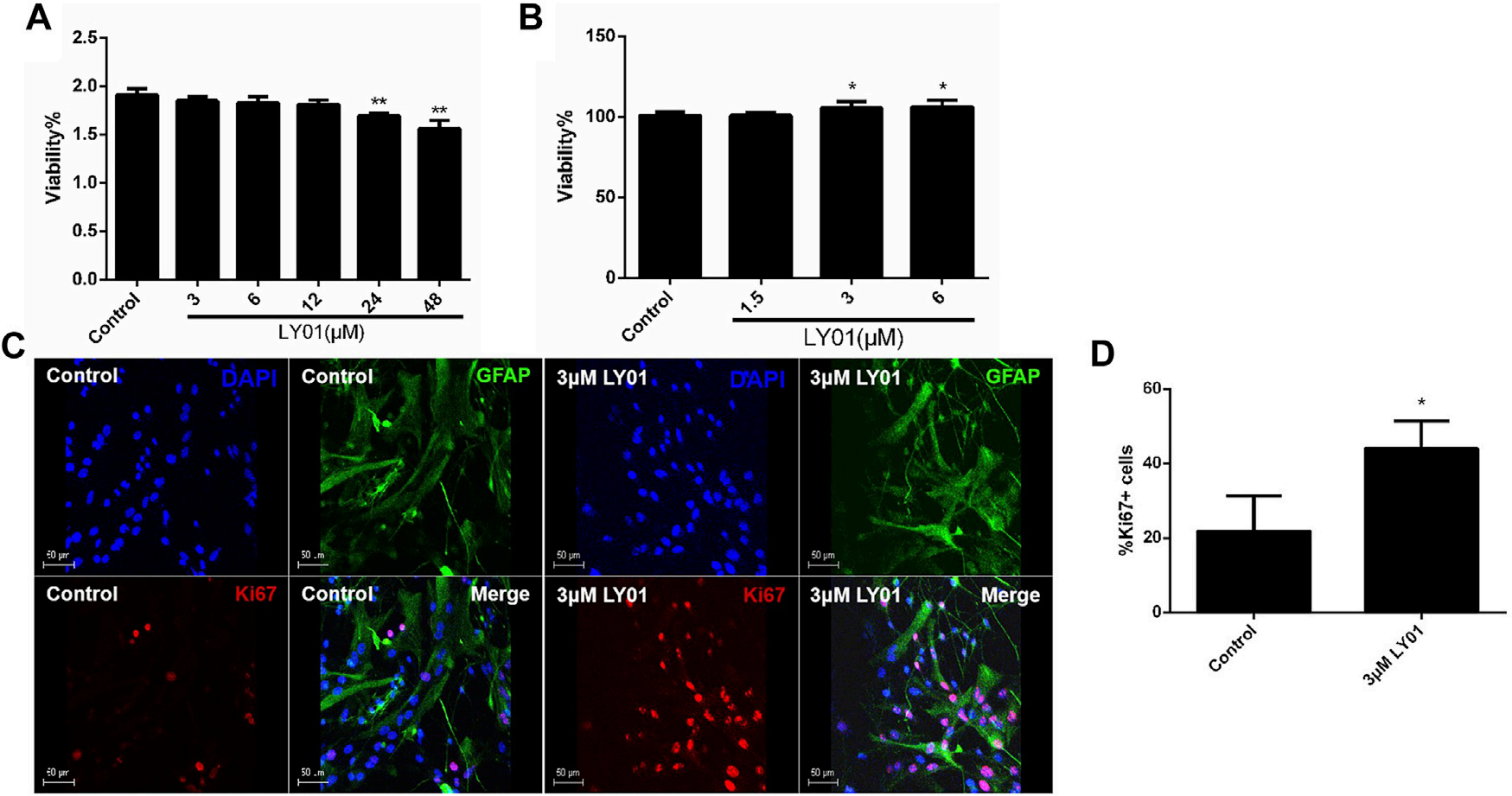
Effects of LY01 on primary hippocampal neurons and primary astrocytes **(A)** Effect of LY01 on the viability of primary hippocampal neurons. Cell viability was detected using water-soluble tetrazolium 1 (WST1); **p < 0.01 vs. Control; *n* = 3 **(B)** Effect of LY01 on the viability of primary astrocytes. Cell viability was detected by WST1; *p < 0.05 vs. Control; *n* = 3 **(C)** Effect of LY01 on astrocyte proliferation. The nuclei are labeled with DAPI (blue), proliferating cells are labeled with Ki67 antibody (red), and neural stem cells (NSCs) are labeled with nestin antibody (green) **(D)** Proportion of cells labeled with both Ki67 and glial fibrillary acidic protein (GFAP) antibodies compared to total GFAP + cells. **p < 0.01 vs. Control; *n* = 3. The results are expressed as means ± SD.

Primary hippocampal neurons cultured *in vitro* (Supplementary Figure S2A) for 3–5 days were divided into six groups: the control group and five experimental groups (3, 6, 12, 24, and 48 μm LY01). After 8 h of treatment, the viability of neurons in the 3, 6, and 12 μm groups had no significant change compared with the control group (*p* > 0.05). The viability of neurons in the 24 and 48 μm groups was lower than that in the control group (*p* < 0.05). Thus, the concentration of LY01 used on neurons in subsequent experiments was less than 24 μm (Figure 7A).

As shown in Figure 7B, primary astrocytes (Supplementary Figure S2B) were treated with 1.5–6 μm LY01 and cell viability was detected 48 h later. It was found that the cell viability was significantly enhanced at concentrations higher than 3 μm (*p* < 0.05). The results of glial fibrillary acidic protein (GFAP) and nuclear protein Ki67 double staining of primary astrocytes treated with 3 μm LY01 showed that LY01 significantly increased the number of new astrocytes and there were approximately double the number of GFAP and Ki67 double-positive cells compared with the control group (Figures 7C,D), indicating that LY01 promoted the proliferation of astrocytes (*p* < 0.05).

### LY01 Promotes Proliferation and Migration of Primary NSCs

**FIGURE 8:**
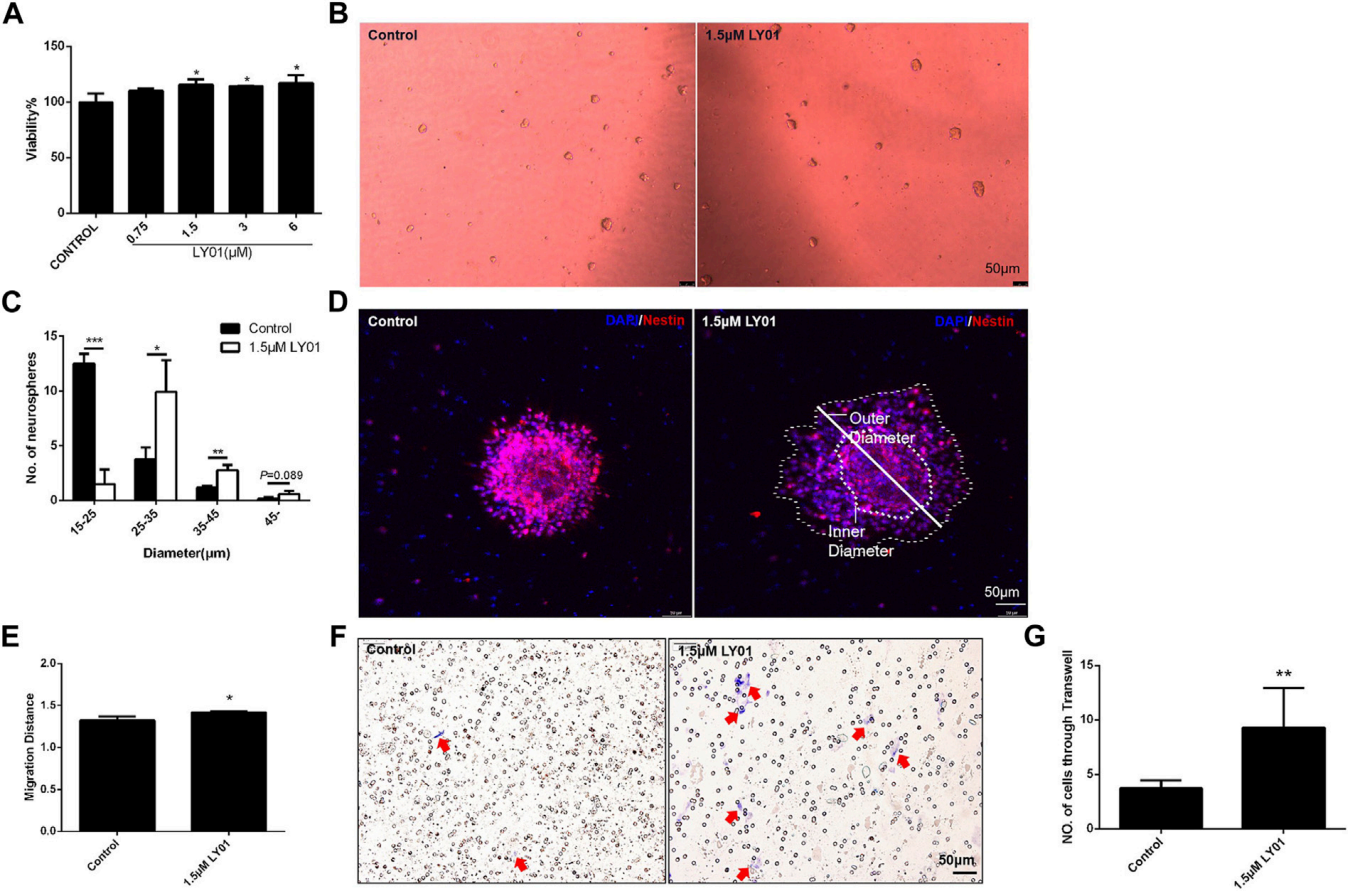
Effects of LY01 on primary NSCs **(A)** Effect of LY01 on the viability of primary NSCs. Cell viability was detected using WST1; *p < 0.05 vs. Control; *n* = 3 **(B)** Neurospheres after LY01 treatment **(C)** Quantification of diameter and quantity of neurospheres after LY01 treatment. *p < 0.05, **p < 0.01, ***p < 0.001 vs. Control; *n* = 3 **(D)** Effect of LY01 on the migration of neurospheres. NSCs are labeled with nestin antibody (red) and the nuclei are labeled with DAPI (blue); the outer and inner diameters are indicated in figure **(E)** Quantification of migration distance of neurospheres. The migration distance is equal to the ratio of the outer diameter after migration to the diameter of the neurosphere (inner diameter). *p < 0.05 vs. Control **(F)** Representative images of Transwell migration assay experiments. The cells that migrated to the chamber below were stained blue with 0.1% crystal violet **(G)** Quantification of Transwell experimental results. *p < 0.05,**p < 0.01 vs. Control. The results are expressed as means ± SD.

The primary NSCs with good proliferation and properties (Supplementary Figures S2C–F) were evenly spread into a 96-well plate for WST1 testing; when the concentration of LY01 reached 1.5 μm, the viability of NSCs was increased (*p* < 0.05, Figure 8A). At concentrations lower than 1.5 μm, the number of NSCs with a diameter of 25–45 μm and the number of clone balls with a diameter greater than 45 μm were significantly higher than those of the control group (*p* < 0.05), indicating that LY01 promoted the proliferation of NSCs (Figures 8B,C).

As shown in Figure 8D, poly-l-lysine induced the adherent neurospheres to migrate radially. The ratio of the diameter of the radiation circle after migration to the diameter of the protoneurospheres was used to reflect the migration ability of stem cells. The results show that LY01 promoted the outward migration of NSCs (Figure 8E). The Transwell migration assay, as a classic method to detect cell migration and invasion, was used to verify the effect of LY01 on the migration of NSCs. As shown in Figure 8F, NSCs in the culture system with 1.5 μm LY01 were twice as large as those in the control group and migrated to the lower layer of the Transwell chamber (Figure 8G), indicating that LY01 promoted the migration of NSCs.

As shown in Supplementary Figure S3A, NSCs differentiated into astrocytes and neuron-like cells after 7 days of induction in DMEM/F12 medium plus FBS (10%). The proportion of GFAP-positive astrocytes and microtubule-associated protein 2 (MAP2)-positive neurons after differentiation indicated that 1.5 μm LY01 had no effect on the selection of cell type during differentiation of NSCs (Supplementary Figure S3B).

### Effects of LY01 on Cell Cycle and Transcription of Primary NSCs

**FIGURE 9:**
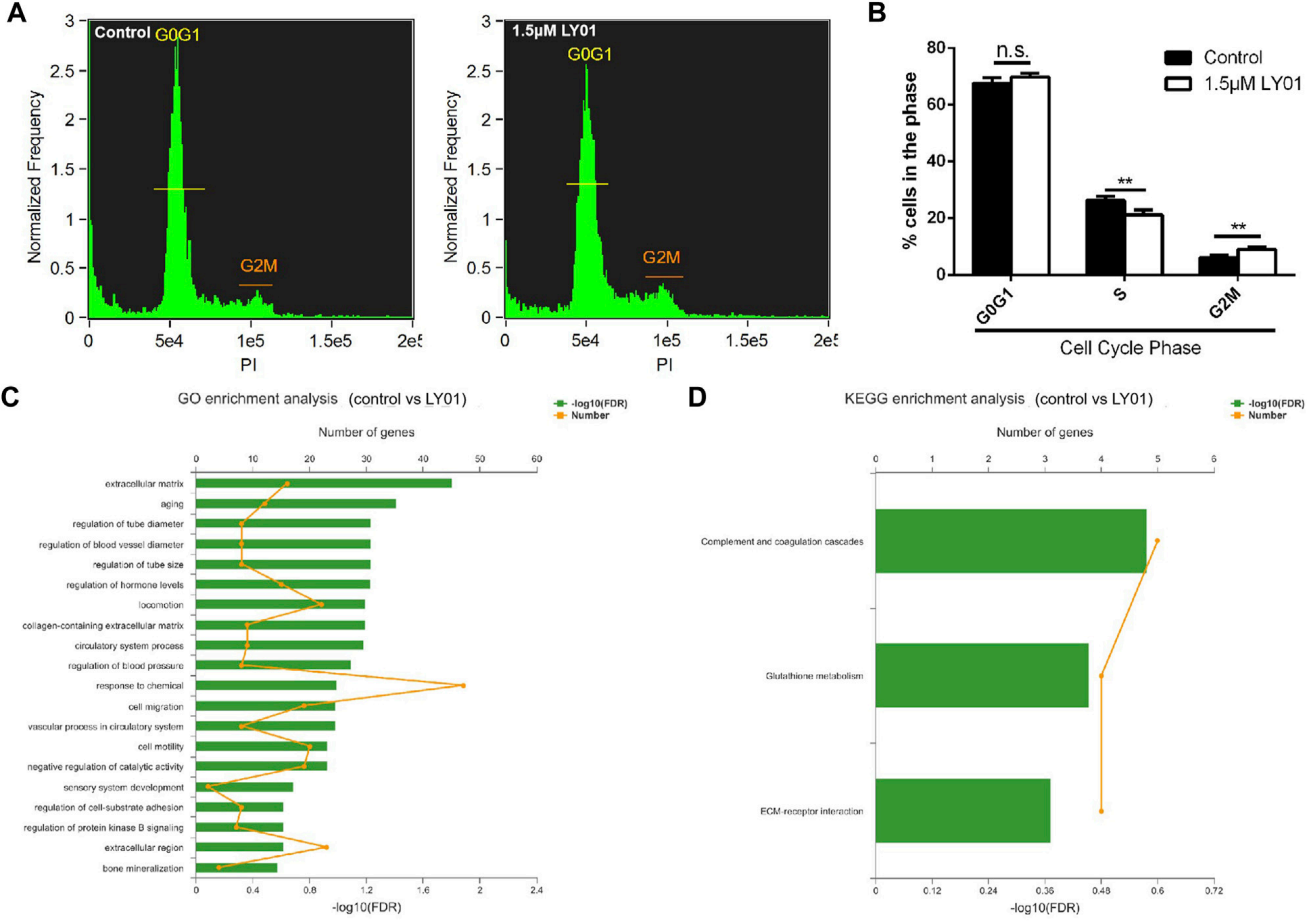
Effects of LY01 on the cell cycle and transcription of primary NSCs **(A)** Cell cycle analysis of primary NSCs after LY01 treatment **(B)** Distribution of NSCs in each cycle. **p < 0.01 vs. Control; *n* = 3 **(C)** Gene Ontology (GO) enrichment analysis **(D)** Kyoto Encyclopedia of Genes and Genomes (KEGG) enrichment analysis. Primary NSCs cultured *in vitro* were treated with 1.5 μm LY01 and the control group was treated with vehicle. After 6 h of treatment, Trizol was used to collect cells and the samples were sent for transcriptome sequencing and analysis. Three parallel samples were set in each group. The results are expressed as means ± SD.

NSCs treated with and without 1.5 μm LY01 were collected. After propidium iodide staining, the distribution of NSCs in each cell cycle was measured using flow cytometry to identify the stage that LY01 regulates the proliferation of NSCs. As shown in Figures 9A,B, μm LY01 treatment induced more cells to enter the G2/M phase through the S phase (*p <* 0.01) but had no effect on the proportion of cells in the G0/G1 phase. These results indicate that LY01 may regulate the S phase of the cell cycle to make more cells enter G2/M, thus promoting NSCs proliferation.

Transcriptome sequencing is an effective method to study the effects of drugs on cells at the transcriptional level. However, combining transcriptome sequencing results with functional enrichment analysis [such as Gene Ontology (GO) and Kyoto Encyclopedia of Genes and Genomes (KEGG) analysis] allows researcher to draw conclusions based on a group of related genes rather than a single gene, which increases the reliability of the study and identifies the biological processes most related to the observed phenomena. GO and KEGG analysis were performed and stored using the online platform of Majorbio Cloud Platform (www.majorbio.com). According to the professional feedback provided by Meggie biological company, the sample quality met the requirements of accurate sequencing and the percentage of the Q30 base was more than 94.66%. A total of 37,464 transcripts were analyzed and detected, including 23,986 known transcripts and 13,478 new transcripts. Among them, 21,456 transcripts were annotated to the GO library, 16,477 transcripts were annotated to the KEGG library, and 237 differentially expressed genes were identified. There were significant differences between the two groups (Supplementary Figure S4). The larger the value of −log_10_ (false discovery rate), the more significant the functional enrichment. Both GO (Figure 9C) and KEGG (Figure 9D) annotation analysis indicated that the extracellular matrix (ECM) and associated receptors may participate in the mechanism of LY01. Lamc2, as a component of the ECM, had the most significant upregulation between the LY01-administered group and the control group (Supplementary Figure S5). In addition, functional enrichment analysis indicated that aging, glutathione metabolism, and other biological processes may also play an important role in the function of LY01. The data for this study have been deposited to the sequence read archive (SRA), and the accession number is PRJNA833033. (https://www.ncbi.nlm.nih.gov/sra/?term=PRJNA833033).

## Discussion

The nervous system is composed of many types of cells, among which neurons, astrocytes, and NSCs play important roles. Neurons integrate incoming information and send out information, effects directly related to human emotion, cognition, and memory changes. Astrocytes are the most widely distributed neural cells in mammals, and their functions include nutrition supply, protection, signal transmission, and inflammatory regulation. Neurotrophic factors secreted by astrocytes at injured sites play an important role in neuronal survival and regeneration. Our study showed that LY01 promotes the proliferation of astrocytes and primary NSCs *in vitro* and increased the number of new neurons in the dentate gyrus of the hippocampus *in vivo*. We speculate that LY01 may promote the proliferation, migration, and differentiation into functional neurons of NSCs in the dentate gyrus area of AD mice under the influence of the microenvironment at the lesion site and maintain the stability of the number of neurons in the hippocampus. Likewise, LY01 promotes the proliferation of hippocampal astrocytes (thus the secretion of more neurotrophic factors) and results in the nutrition supply and protection of neurons.

By analyzing the results of male and female mice after administration of LY01, we found that although LY01 has estrogen like structure, intravenous injection of LY01 will not cause obvious side effects on mice of both genders due to excessive estrogen, and both showed obvious effect of promoting neural regeneration. As natural plant products have fewer or no side effects and are easily available, their effect of AD treatment has been widely studied (Varshney and Siddique, 2021). But most of their mechanisms focus on neuroprotection, antioxidation, acetylcholinesterase inhibition and Aβ deposition reduction (Varshney and Siddique, 2021; Park et al., 2014). This study enriches the mechanism of natural products in the AD prevention or treatment. Meanwhile, most studies on the prevention or treatment of AD by promoting endogenous neural regeneration are about growth factors (Hassouna et al., 2016; Vasic et al., 2019), and a few of these studies have been conducted in AD animal models (Cevik et al., 2017). Among them, there are few studies on natural plant products. Therefore, this study will have great reference value for the exploration of natural products in the treatment of AD through neural regeneration mechanism in 5×FAD mice. In recent years, more researchers began to pay attention to the important role of neuron loss in AD, the results of this study support the hypothesis of prevention or treatment of AD by promoting endogenous hippocampal neural regeneration (Oakley et al., 2006; Eimer and Vassar, 2013). However, many therapies have failed in clinical trials (Cummings et al., 2019) in patients with established AD, suggesting that, once developed, disease-modifying agents may need to be deployed earlier in the course of illness (Hort et al., 2010; Grossberg et al., 2019). According to the results of this study, the strategy of treatment at a early stage of AD is feasible, which supports this assumption. However, there are some deficiencies, such as it is impossible to obtain the difference of drug response between male and female mice under the same administration conditions. But this problem can be a subject of follow-up research.

Transcriptome sequencing revealed that the proliferation and migration of astrocytes and NSCs promoted by LY01 may be related to the regulation of the ECM and associated receptors, such as Lamc2. The ECM includes insoluble structural components, such as collagen and glycoprotein, and proteinases and cytokines related to matrix metabolism (Wilems et al., 2019). Some studies have shown that laminin is the main type of glycoprotein in the ECM and can promote adhesion and regulate cell proliferation and migration to repair brain injury (Qian et al., 2018; Ma et al., 2020), consistent with the functional research results of this study. However, this study lacks direct experimental evidence on how ECM and associated receptors participate in LY01 induced neural regeneration. And the up regulation of Lamc2 detected in this study has been reported to be related to the occurrence of a variety of cancers (Yamamoto et al., 2009; Zhang et al., 2018; Jing et al., 2020). Thus, whether LY01 has carcinogenicity and other side effects need to be further studied.

Because of the earlier administration of LY01 in this study’s experimental design, some of the conventional behavioral indicators of cognitive and memory decline were not significant reflections of the effects of the drug. The behavioral test data collected from 5×FAD mice were highly variable and thus not conducive to reflect sensitively the effects of LY01. The positive control mice in Rg1 group also did not show significant cognitive and memory improvement in the behavioral tests. According to the current researches, the potential mechanisms, by which Rg1 significantly improved cognitive behavioral impairments in most Alzheimer’s disease models, included antioxidant and anti-inflammatory effects, amelioration of Alzheimer’s disease-related pathology, synapse protection, and up-regulation of nerve cells via multiple signaling pathways (Liang et al., 2021). However, considering Alzheimer’s disease is a multi-mechanism disease, we speculate that the main mechanism regulated by Rg1 is not the essential mechanism causing dementia-like behaviors in the early stage of 5×FAD mice in this study. In addition, the results of BrdU and DCX staining indicated that for the 13-week-old 5×FAD mice, the number of new cells and the number and neurite length of neural precursor cells in the dentate gyrus area were significantly lower than those in WT mice, indicating that the abnormal neural regeneration ability and neural cell morphology may be the earlier pathological changes of AD.

The first clinical symptom of AD is the decline of short-term memory; the decline of neural regeneration in patients with AD affects the number and functional maintenance of neurons and subsequently affects the construction of new short-term memory loops in the neural network. Therefore, maintaining the number and vitality of regenerated neural cells in the brains of patients with AD is an important idea in the treatment of AD. There is no clinical drug for AD patients that targets the endogenous neural regeneration process. LY01 has shown to play a significant role in promoting endogenous neural regeneration, and thus is expected to fill this gap.

## Conclusion

All these findings together demonstrated that LY01 could reduce the decline of cognition and memory in the early stage of 5×FAD mice by regulating the extracellular matrix and improving neuronal regeneration in the hippocampus. It induced the proliferation of astrocytes, the proliferation and migration of NSCs, and increased the number of new cells and neural precursor cells in the dentate gyrus area of 5×FAD mice. This phenomenon could be observed both in female and male 5×FAD mice after LY01 treatment for more than 2 weeks. And after 5-weeks LY01 treatment, the spatial and conditioned memory abilities were significantly improved in 5×FAD mice according to the results of the Morris water maze and fear conditioning test. The neuronal regeneration induced by LY01 was related to the regulation of the extracellular matrix, associated receptors, and the S phase of the cell cycle. LY01 promoted the regeneration of neuronal cells and alleviates the symptoms of AD in the early stage, which provided new ideas and alternative drugs for the AD prevention and treatment.

## Data Availability

The datasets presented in this study can be found in online repositories. The names of the repository/repositories and accession number(s) can be found in the article/Supplementary Material.
