# Supplementary figures and images for: Mechanism of Neural Regeneration Induced by Natural Product LY01 in the 5×FAD Mouse Model of Alzheimer’s Disease
Source: Front Pharmacol. 2022 Jun 22;13:926123. doi: 10.3389/fphar.2022.926123 (PMC9258960; doi:10.3389/fphar.2022.926123)

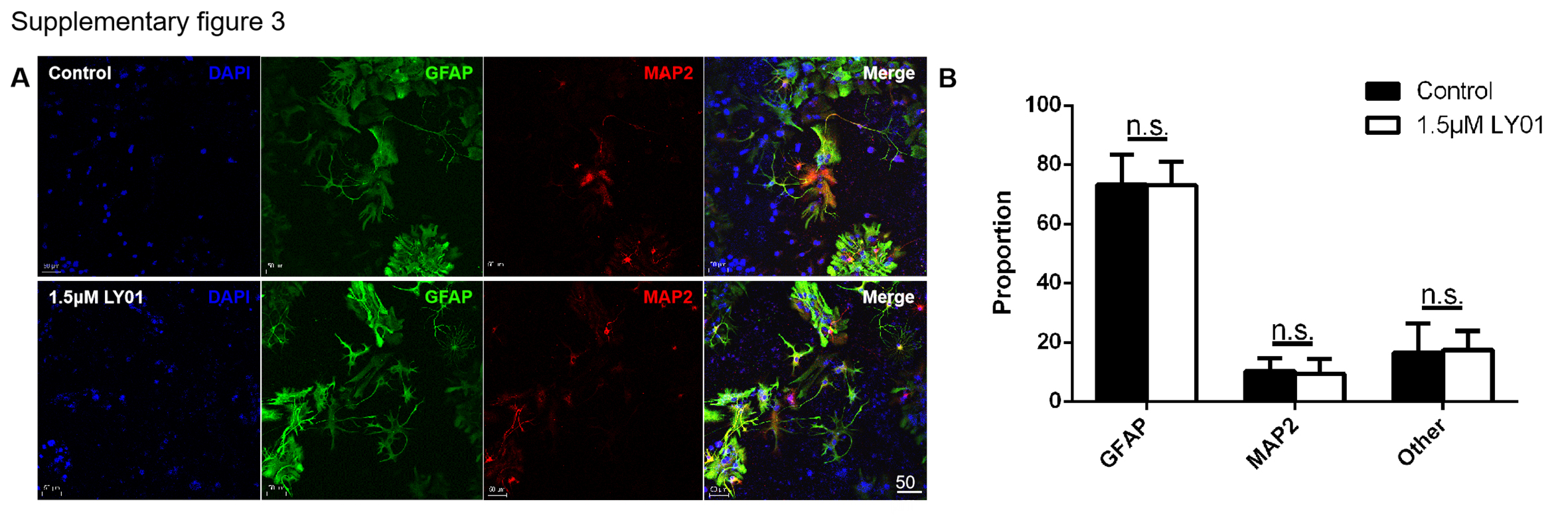

Supplement: Supplementary file 1 [file Image3.JPEG]

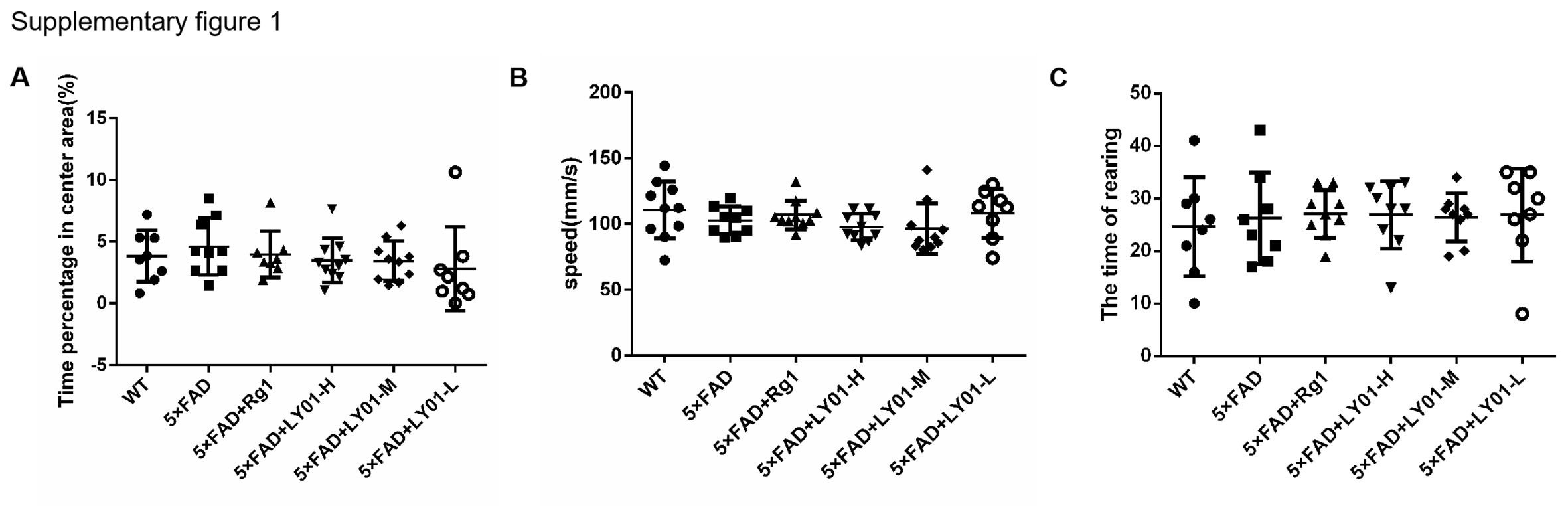

Supplement: Supplementary file 2 [file Image1.JPEG]

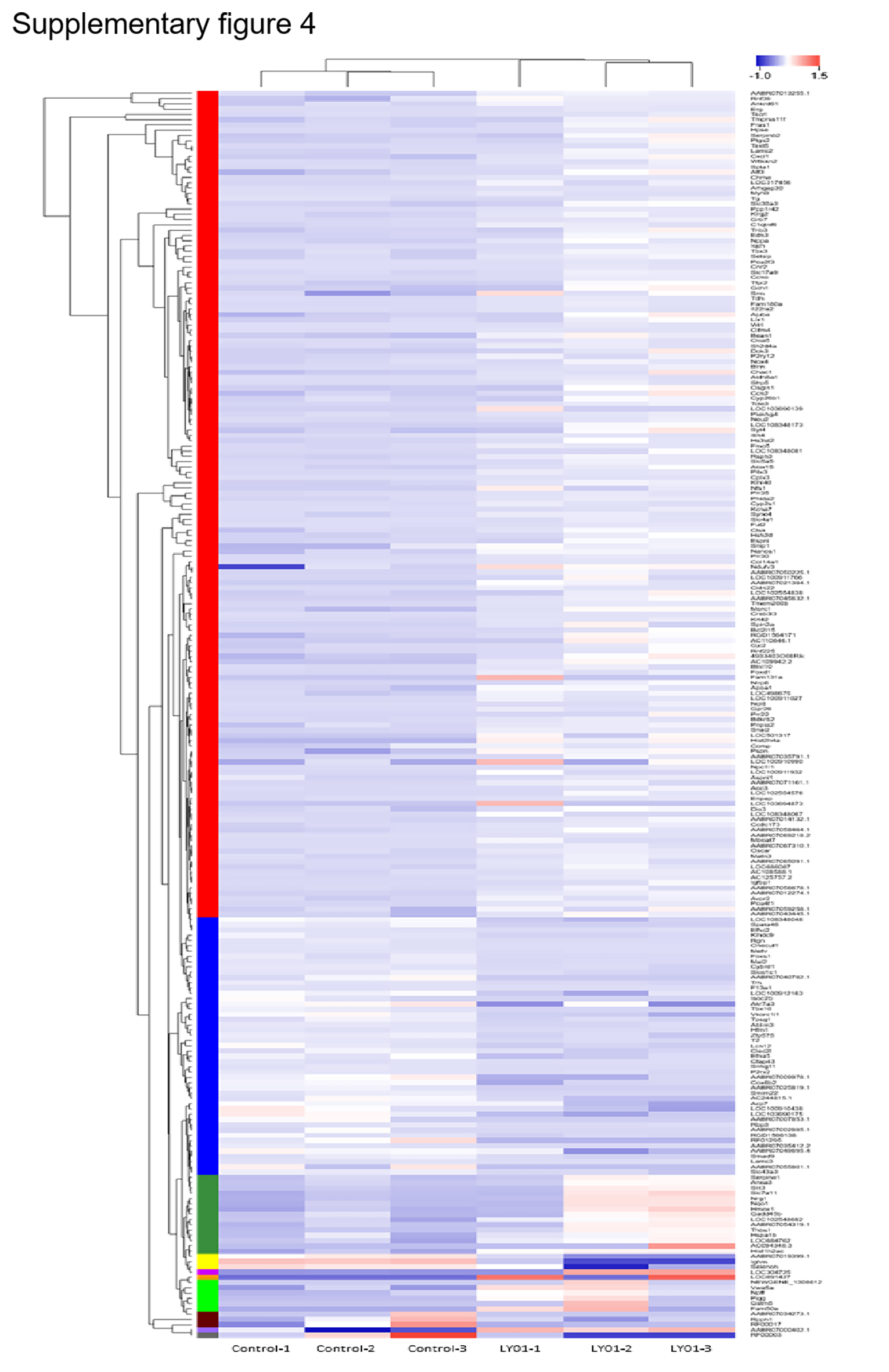

Supplement: Supplementary file 3 [file Image4.JPEG]

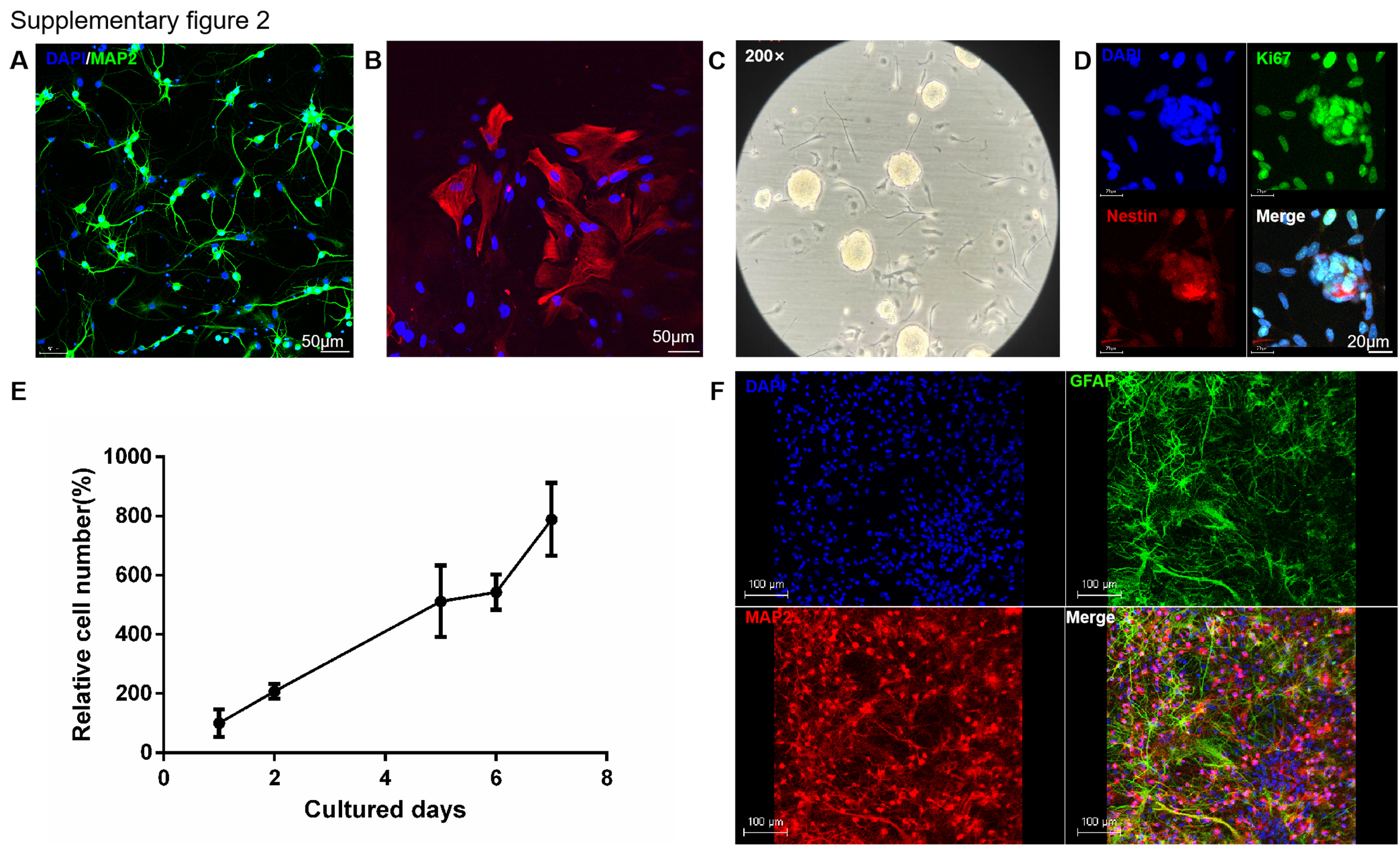

Supplement: Supplementary file 4 [file Image2.JPEG]

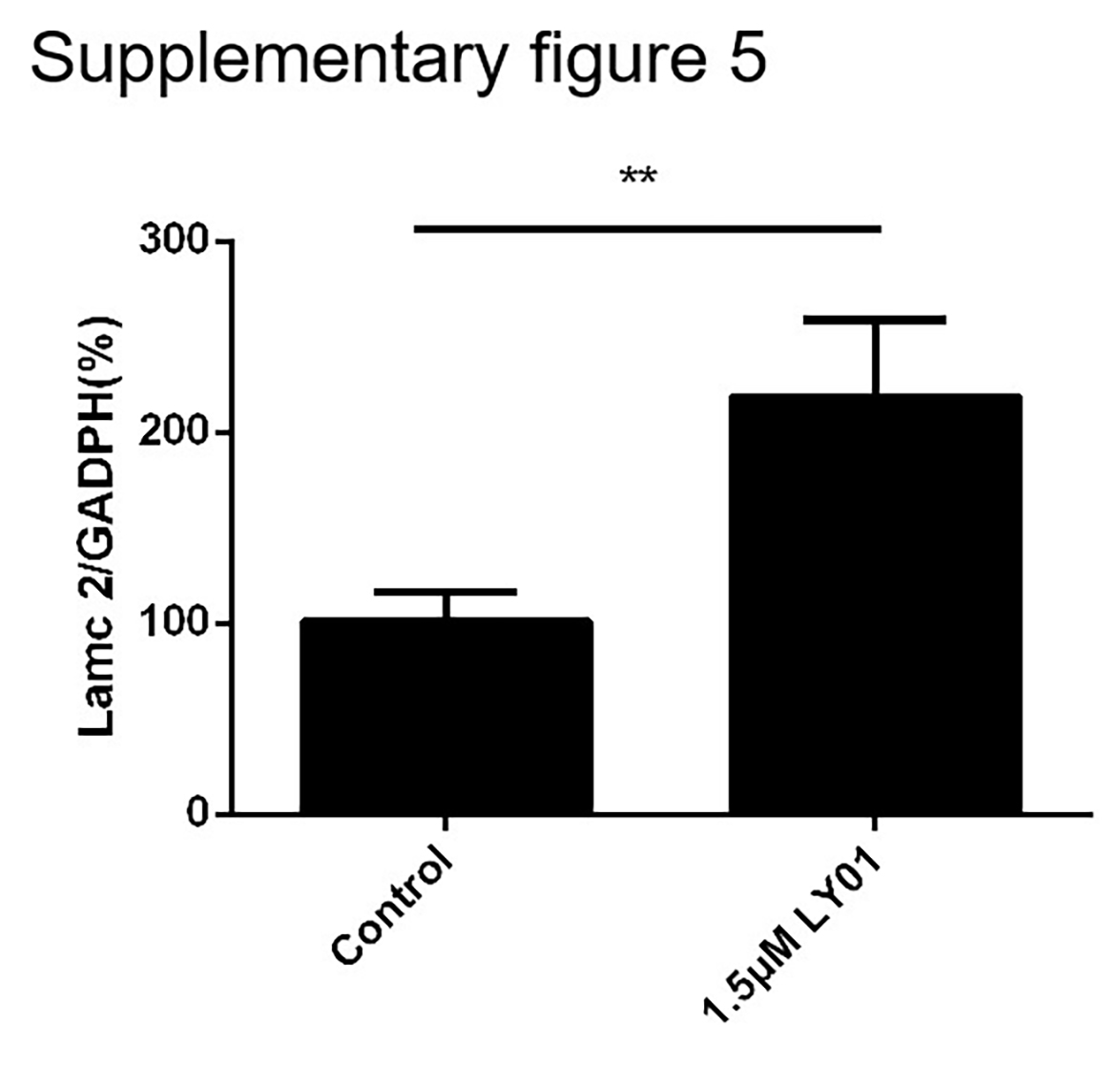

Supplement: Supplementary file 5 [file Image5.JPEG]
